# The Prince of Wales's Hospital Fund

**Published:** 1897-11-27

**Authors:** 


### THE PRINCE OF WALES'S HOSPITAL FUND.

The stamp books issued by the organisers of the Prince of Wales's Hospital Fund are now on sale. These books fulfil the two-fold purpose of conserving the stamps in a convenient and appropriate manner, and of recording in prominent form subscriptions to the hospitals. The little volume is beautifully got up, attractively bound in red cloth, and is procurable for the small sum of sixpence, so that it is within reach of all.

